# Manipulation of ZDS in tomato exposes carotenoid‐ and ABA‐specific effects on fruit development and ripening

**DOI:** 10.1111/pbi.13377

**Published:** 2020-04-20

**Authors:** Ryan P. McQuinn, Nigel E. Gapper, Amanda G. Gray, Silin Zhong, Takayuki Tohge, Zhangjun Fei, Alisdair R. Fernie, James J. Giovannoni

**Affiliations:** ^1^ Department of Plant Biology Cornell University Ithaca NY USA; ^2^ Boyce Thompson Institute for Plant Research Cornell University Ithaca NY USA; ^3^ Max‐Planck‐Institut fur Molekulare Pflanzenphysiologie Potsdam‐Golm Germany; ^4^ Robert W. Holley Center for Agriculture and Health USDA‐ARS Cornell University Ithaca NY USA; ^5^ Present address: Australian Research Council Centre of Excellence in Plant Energy Biology Research School of Biology The Australian National University Canberra ACT 2601 Australia

**Keywords:** fruit ripening, fruit development, carotenoid biosynthesis, zeta‐carotene desaturase, *Solanum lycopersicum*, abscisic acid

## Abstract

Spontaneous mutations in fruit‐specific carotenoid biosynthetic genes of tomato (*Solanum lycopersicum*) have led to improved understanding of ripening‐associated carotenogenesis. Here, we confirm that *ZDS* is encoded by a single gene in tomato transcriptionally regulated by ripening transcription factors RIN, NOR and ethylene. Manipulation of ZDS was achieved through transgenic repression and heterologous over‐expression in tomato. CaMV 35S‐driven RNAi repression inhibited carotenoid biosynthesis in all aerial tissues examined resulting in elevated levels of *ζ*‐carotene isomers and upstream carotenoids, while downstream *all trans‐*lycopene and subsequent photoprotective carotenes and xanthophylls were diminished. Consequently, immature fruit displayed photo‐bleaching consistent with reduced levels of the photoprotective carotenes and developmental phenotypes related to a reduction in the carotenoid‐derived phytohormone abscisic acid (ABA). *ZDS*‐repressed ripe fruit was devoid of the characteristic red carotenoid, *all trans‐*lycopene and displayed brilliant yellow pigmentation due to elevated 9,9′ *di‐cis‐ζ*‐carotene. Over‐expression of the *Arabidopsis thaliana ZDS* (*AtZDS*) gene bypassed endogenous co‐suppression and revealed ZDS as an additional bottleneck in ripening‐associated carotenogenesis of tomato. Quantitation of carotenoids in addition to multiple ripening parameters in ZDS‐altered lines and ABA‐deficient fruit‐specific carotenoid mutants was used to separate phenotypic consequences of ABA from other effects of ZDS manipulation and reveal a unique and dynamic *ζ*‐carotene isomer profile in ripe fruit.

## Introduction

Carotenoids play pivotal roles throughout plant development, among the most observable being coloration of ripe fruit and flowers for attraction of seed dispersing frugivores and pollinators. Carotenoids are additionally requisite to maintain proper function of the photosynthetic apparatus. Photoprotective carotenes (i.e. β‐carotene) and xanthophylls (i.e. lutein, zeaxanthin, violaxanthin and neoxanthin) are localized in photosystems I and II where they absorb excess light energy and limit damage from adjacent excited chlorophylls through quenching of reactive singlet oxygen (Amunts *et al.*, 2007; Jahns and Holzwarth, 2012). Numerous aspects of plant development, dormancy and stress responses are dependent on the synthesis of the carotenoid‐derived phytohormone abscisic acid (ABA) (Cutler *et al.*, 2010; Hauser *et al.*, 2011; Mauch‐Mani and Mauch, 2005; Vishwakarma *et al.*, 2017; Zhang *et al.*, 2006). Recent evidence indicates additional roles of carotenoids as precursors for important regulatory functions via strigolactones (reviewed in Waters *et al.*, 2017) in addition to signalling functions with other organisms through volatile carotenoid metabolites (reviewed in Hou *et al.*, 2016; McQuinn *et al.*, 2015).

Utilization of available natural tomato (*Solanum lycopersicum*) carotenoid biosynthetic mutants in combination with studies in other model species (e.g. *Arabidopsis thaliana* and *Zea mays*) elucidated a nearly comprehensive carotenoid biosynthetic pathway (Figure S1). Moreover, duplication of genes in a majority of biosynthetic steps in tomato illuminated chloroplast‐ and chromoplast‐specific pathways clarifying ripening‐associated carotenogenesis (Galpaz *et al.*, 2006; Sato *et al.*, 2012; Figure S1 and Table S1). Natural mutations in genes involved in chromoplast‐specific carotenogenesis (e.g. *r, PSY1*; *tangerine*, *CRTISO*; *Beta*, *CYC‐B/CRTL‐B2*) have allowed researchers to functionally define many steps of the pathway relevant to fruit ripening (Fray and Grierson, 1993; Isaacson *et al.*, 2002; Ronen *et al.*, 2000) and led to some of the colour variation in tomato (yellow and orange fruit varieties) appreciated by consumers. However, the lack of gene duplication within the poly‐*cis*‐transformation of 15‐*cis*‐phytoene to *all trans*‐lycopene limits the assessment of ripening roles for these genes.

In the poly‐*cis*‐transformation of 15 *cis*‐phytoene to *all trans*‐lycopene, 15‐*cis*‐phytoene undergoes four desaturation steps carried out by two desaturases (phytoene desaturase/PDS and zeta‐carotene desaturase/ZDS) in conjunction with two intermediary isomeric conversions carried out by two isomerases (zeta‐carotene isomerase/ZISO and carotenoid isomerase/CRTISO; Figure S1; Beltrán *et al.*, 2015; Brausemann *et al.*, 2017; Chen *et al.*, 2010b; Dong *et al.*, 2007; Isaacson *et al.*, 2002; Qin *et al.*, 2007). Unlike ZISO and CRTISO which carry out reactions that can alternatively be catalysed by light in photosynthetic tissues (Isaacson *et al.*, 2002; Li *et al.*, 2007, respectively), mutations in the single copy genes PDS and ZDS manifest in dramatically reduced plant fitness reflecting highly unstable photosynthetic tissues (Dong *et al.*, 2007; Qin *et al.*, 2007). This is likely the reason that, until recently, PDS and ZDS remained uncharacterized in terms of tomato fruit ripening (McQuinn *et al.*, 2017). Initial characterization of PDS and ZDS was limited to their transient repression via virus‐induced gene silencing (VIGS) due to their indispensable nature in photosynthetic tissues (Fantini *et al.*, 2013). More recently, we further characterized PDS via heterologous over‐expression of the *Arabidopsis PDS* in tomato and investigated its impact on the carotenoid accumulation and gene expression throughout the aerial tissues of the tomato plant (McQuinn *et al.*, 2017).

Genes involved in synthesis of 15 *cis‐*phytoene and its poly‐*cis*‐transformation to *all trans*‐lycopene are induced transcriptionally at the onset of fruit ripening giving the tomato its characteristic red colour. It is well established that the initiation of physiological changes associated with tomato ripening, including carotenoid accumulation, is induced by a climacteric burst of ethylene, and is dependent on an elaborate combination of epigenetic and transcriptional dynamics influenced to varying degrees by ripening‐specific transcriptional regulators RIN (a MADS‐box transcription factor), NAC‐NOR (a NAC domain transcription factor) and CNR (a Squamosa promoter binding protein; Eriksson *et al.*, 2004; Gallusci *et al.*, 2016; Giovannoni *et al.*, 2017; Giovannoni *et al.*, 2004; Ito *et al.*, 2017; Lü *et al.*, 2018; Manning *et al.*, 2006; Vrebalov *et al.*, 2002).

As the main rate‐limiting step of carotenogenesis, phytoene synthase (PSY) has garnered much attention regarding its transcriptional and post‐transcriptional regulation across various plant species (i.e. Arabidopsis, Zhou *et al.*, 2015; Melon, Chayut *et al.*, 2017). This holds true in tomato, where *PSY1* has been well studied and shown to be transcriptionally regulated by ethylene perception and signalling (Gapper *et al.*, 2013), and a direct target of RIN*‐*mediated transcriptional regulation (Martel *et al.*, 2011), which remains dependent on demethylation of its promoter prior to ripening (Zhong *et al.,* 2013). Further, numerous strategies targeting PSY1 have been deployed to enhance carotenoid accumulation in tomato (reviewed in Fraser *et al.*, 2009). Recently, researchers enhance carotenoid accumulation in tomato upon ectopic expression of the *Arabidopsis* ORANGE protein, which has dual roles in post‐transcriptionally regulating PSY as well as inducing chromoplast biogenesis (Yazdani *et al.*, 2019). While such strategies have proven successful, new bottlenecks emerge in subsequent desaturase steps carried out by PDS and ZDS, thereby limiting their effectiveness. These secondary bottlenecks present additional targets for manipulation to enhance carotenoid content, one of which, PDS, has been successfully exploited to increase downstream health‐promoting carotenoids demonstrating its potential in carotenoid biotechnology strategies (McQuinn *et al.*, 2017).

Regarding the enzymes carrying out the poly‐*cis‐*transformation of 15‐*cis‐*phytoene to *all trans‐*lycopene, very little is known regarding their regulation, other than their induction by ethylene perception and signalling upon initiation of tomato fruit ripening (Alba *et al.*, 2005). Further, while ZISO and CRTISO have been characterized via natural mutations (Gonda *et al.*, 2019; Isaacson *et al.*, 2002, respectively) and now PDS through transgenic approaches (Fantini *et al.*, 2013; McQuinn *et al.*, 2017), ZDS remains the least characterized step in tomato fruit ripening‐associated carotenogenesis. Therefore, transgenic manipulation of ZDS and exploration into its regulation is essential and may aid future strategies aimed at enhancing carotenoid content beyond levels achieved via PSY exploitation.

Given the limited knowledge of ZDS in terms of its regulation and phenotypic consequences on fruit development and ripening, we explored the consequences of transgenic manipulation of ZDS throughout tomato fruit development, investigating its function via RNAi‐guided repression and over‐expression using a heterologous gene from Arabidopsis (*AtZDS*). The heterologous over‐expression of *AtZDS* was implemented to reduce potential co‐suppression of the endogenous tomato *ZDS* gene, a strategy that has proven useful for the manipulation of *PDS* in tomato (McQuinn *et al.*, 2017). Herein, we identify *ZDS* as a heavily regulated gene in carotenogenesis by multiple ripening regulatory factors. Additionally, we highlight the consequences of ZDS manipulation on fruit ripening carotenogenesis and its potential for the enhancement of carotenoid content in tomato fruit. Lastly, we confirm diminished ABA and carotenoid accumulation throughout tomato fruit development reduces fruit growth and expansion, delays the initiation of fruit ripening and alters fruit quality (i.e. primary metabolites and brix^o^ content).

## Results

### *ZDS* is a single copy gene in tomato strongly regulated by ripening transcription factors and ethylene

In tomato, multiple steps of carotenoid biosynthesis are represented by multigene families (e.g. *Solanum lycopersicum PSY1*; *SlPSY2*; *SlPSY3*, and *SlLCY‐B/ CRTL‐B1*; *SlBCYC/ CRTL‐B2*; Table S1), the origins of which can be traced in some cases to a whole‐genome triplication event Tomato Genomics Consortium (TGC, 2012). These genes have been further categorized according to chloroplast and chromoplast specificity (Table S1; Galpaz *et al.*, 2006). Chromoplast‐ or fruit ripening‐specific genes have been functionally characterized in tomato through the study of natural mutations that have minor effects, if any, on non‐fruit photosynthetic tissues. Given that a tomato *ZDS* mutant has not yet been reported, and consistent with the many deleterious phenotypes of *Arabidopsis* and *Zea mays ZDS* mutants, it is likely that *ZDS* is represented by a single copy gene in tomato and many other widely studied plant species. Queries of available protein sequences using tomato and *Arabidopsis* ZDS (SlZDS and AtZDS, respectively) as the query in multiple sequence/genome databases (NCBI, http://www.ncbi.nlm.nih.gov; TAIR, www.arabidopsis.org; Solgenomics, Solgenomics.net) indicates this is the case for tomato and many other plant genomes. It is noteworthy that genome duplication in apple and orange may have led to an increase from one to two or more paralogous copies of *ZDS*, respectively, in these lineages (Chen *et al.*, 2010a; Velasco *et al.*, 2010).

**Figure 1:**
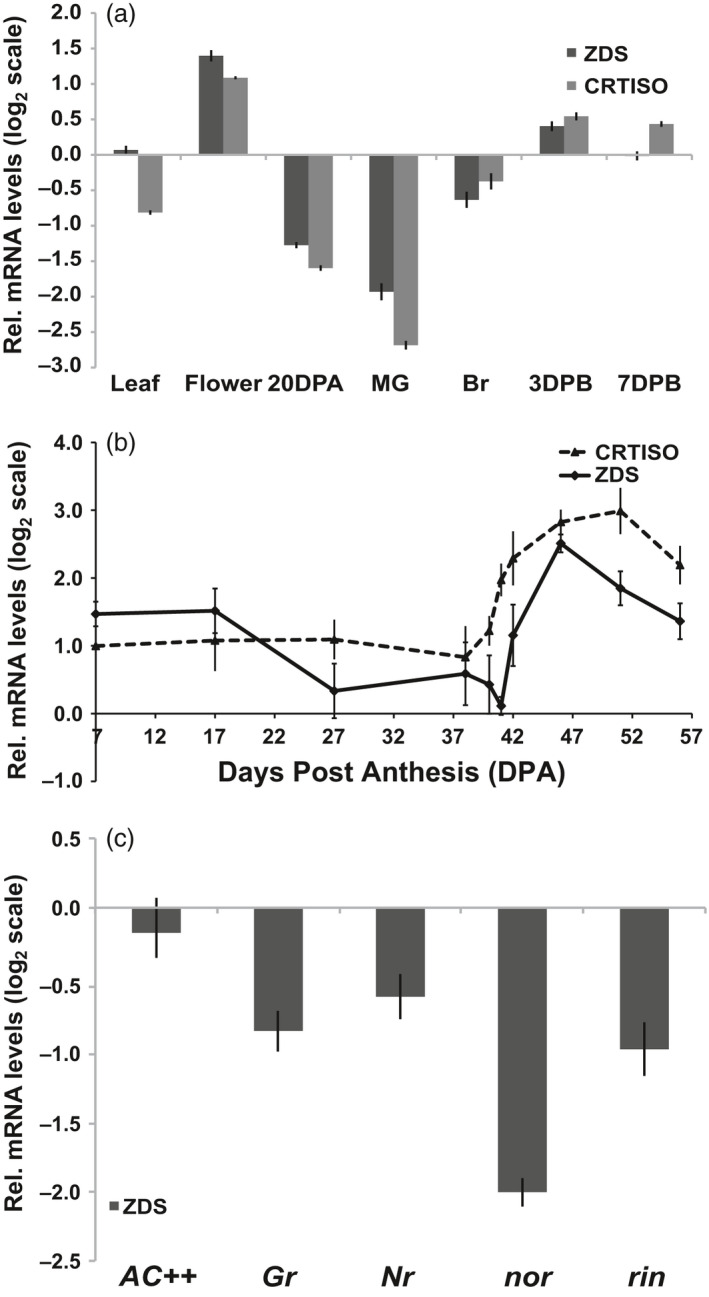
Tomato *ZDS* and *CRTISO* gene expression. (a) mRNA levels in aerial tissues in wild‐type (*AC++*). 20DPA, 20 days post‐anthesis; MG, mature green; Br, breaker; 3DPB and 7DPB, 3 and 7 days post‐breaker. (b) mRNA levels during wild‐type (*AC++*) fruit development. Fruit stages analysed are 7, 17, 27 DPA, MG (38 DPA), Br‐1day (40 DPA), Br (41 DPA), 1, 5, 10 and 15 DPB. (c) ZDS mRNA levels in ripening mutants compared to wild‐type (*AC++*). *rin, ripening inhibitor; nor, non‐ripening; Nr, Never ripe; Gr, Green ripe.* Each stage, tissue and genotype analysed were represented by 3 biological replicates done in triplicate.

Analysis of *ZDS* transcript accumulation in above ground tissues of tomato via qRT‐PCR demonstrates *ZDS* is expressed in all tissues analysed, consistent with available microarray and RNA‐seq data (TGC, 2012; Fernandez‐Pozo *et al.*, 2017; Shinozaki *et al.*, 2018; Zouine *et al.*, 2017; ted.bti.cornell.edu). *ZDS* was most highly expressed in the leaves, ripening fruit and flowers, in increasing order (Figure 1a). 9,9′ *di‐cis*‐ζ‐carotene is converted to *all trans‐*lycopene by ZDS in conjunction with carotenoid isomerase (CRTISO), whose expression pattern for the most part paralleled *ZDS* (Figure 1a). *ZDS* expression analysis through a comprehensive fruit developmental time course reveals *ZDS* transcripts accumulate during early stages of fruit development, after which *ZDS* transcript levels decreased until the onset of ripening at which point expression increases substantially through early ripening (Figure 1b).

Because *ZDS* expression is induced upon ripening initiation, *ZDS* transcript levels were quantified in tomato ripening transcription factor (*ripening inhibitor, rin,* and *non‐ripening, nor*) and ethylene insensitive (*Green ripe, Gr, and Never ripe, Nr*) mutants at the 7 days post‐breaker (7DPB) stage (Barry *et al.*, 2005; Giovannoni *et al.*, 2004; Hackett *et al.*, 2000; Vrebalov *et al.*, 2002). For accurate fruit staging, wild‐type and mutant flowers were all tagged at anthesis and ripening mutant fruits were collected at the same number of days after pollination as that of 7DPB wild‐type fruit where ‘breaker’ was determined by the first sign of colour change. *ZDS* transcripts are reduced in all four mutants with the greatest reduction observed in *nor* fruit. These results highlight NOR, a NAC domain transcription factor, as influential in the regulation of ZDS, and remain consistent with the RIN transcription factor and ethylene all as ripening‐related *ZDS* regulatory components (Figure 1c).

### *ZDS*‐*RNAi* lines demonstrate inhibited carotenogenesis in flowers and fruits

**Figure 2:**
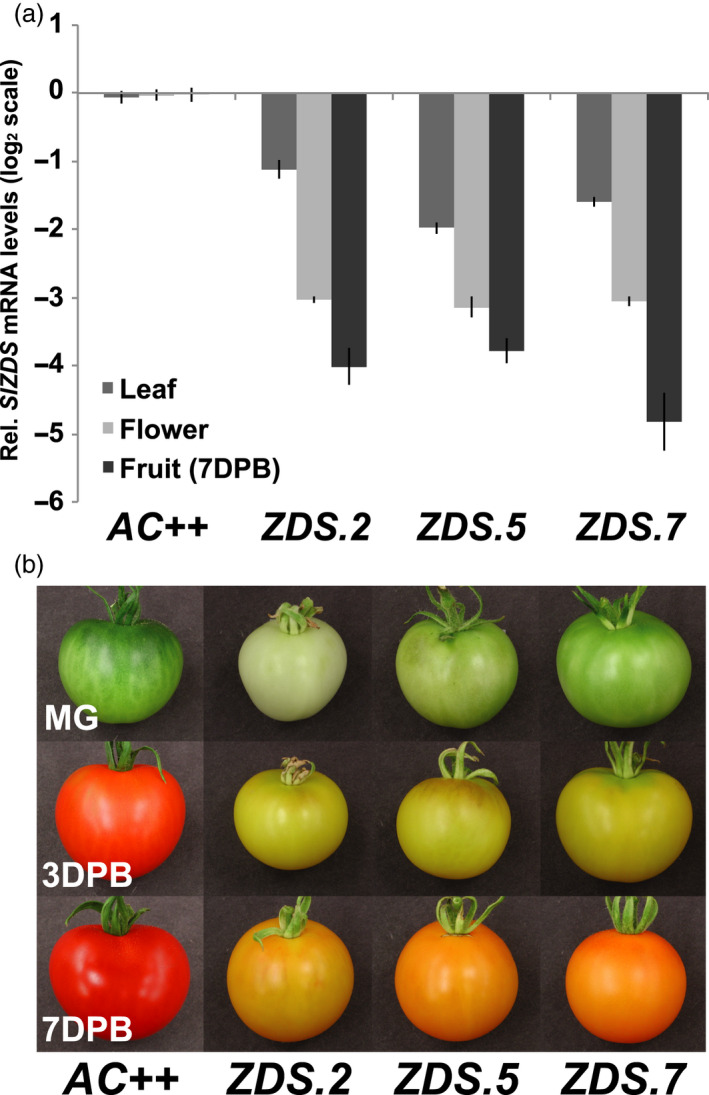
Phenotypic characterization of tomato fruit from ZDS‐RNAi lines. (a) Tissue‐specific repression of ZDS mRNA levels in ZDS‐RNAi lines relative to wild‐type (*AC++)* (*n* = 3 and performed in triplicate). (b) Visually apparent alteration of chlorophyll and carotenoid content during fruit development in ZDS‐RNAi lines compared to wild‐type (*AC++*). MG, mature green; BR, breaker; RR, red ripe. Photographs are not to scale and are relevant only to fruit pigmentation.

ZDS expression was suppressed via RNA interference driven by the constitutive CaMV 35S promoter in the pHELLSGATE2 vector containing cDNA targeting the gene‐specific 3’UTR of *ZDS*. *Agrobacterium tumefaciens*‐mediated transformation of wild‐type tomato (*S. lycopersicum* cv Ailsa Craig) yielded eight independent transgenic *ZDS‐RNAi* lines, three of which (*ZDS.2, ZDS.5* and *ZDS.7*) were selected for further characterization based on insert number, and limited deleterious effects on photosynthetic tissues ensuring flower and fruit set (Figure S2). A number of chlorotic RNAi lines were removed from the analysis due to their inability to propagate. Quantitative PCR confirmed lines *ZDS.5* and *ZDS.7* as single insertion lines, whereas *ZDS.2* contains two transgene insertions. Plants heterozygous for both inserts (in the case of *ZDS.2*) and for the single insert in lines *ZDS.5* and *ZDS.7* were used for subsequent characterization as homozygous plants were severely dwarfed and incapable of proliferation and propagation (Figure S3). RNAi resulted in significant repression of *ZDS* in young leaves, anthesis flowers and ripe fruits of the three independent RNAi lines as compared to wild‐type and all three lines displayed phenotypes consistent with altered carotenogenesis (Figure 2). The strongest repression of *ZDS* was observed in anthesis flowers (~8‐fold decrease) and in ripe fruit (~16‐fold decrease), while expression was repressed approximately twofold in leaves of repression lines (Figure 2a).

Leaves of two‐month‐old plants began to photobleach when removed from low light conditions and placed in natural light (Figure S2). Interestingly, this susceptibility to photobleaching was temporary, as newly formed leaves became visually indistinguishable when compared to wild‐type throughout the remainder of the growing cycle. Similar results were reported by Dong *et al. *(2007) for the weaker *Arabidopsis ZDS*‐mutant allele of *spontaneous cell death 1* (*spc1‐1)* when grown in short‐day conditions. These observations are consistent with photoprotective carotenes and xanthophylls accumulating to wild‐type levels in the leaves of the *ZDS‐RNAi* lines (Table 1). Further, given the accumulation of β‐carotene and xanthophylls remains unchanged in leaves, it is likely that carotenoid‐derived hormones, strigolactone and ABA are not substantially impacted in those tissues as well.

**Table 1:** Carotenoid content in young leaves, anthesis flowers, and ripe fruits of ZDS‐RNAi lines compared to wildtype (Ailsa Craig) (*n*≥3)

Genotype	Total Phytoene	Total Phytofluene	Total □‐carotene	Total Lycopene	Total β‐carotene	Lutein	Other Xanthophylls	Total Carotenoids
Leaf								
*AC++*	0.1 (0.01)	n.d.			102.8 (2.26)	168.2 (6.27)	79.8 (5.23)	357.9 (13.15)
*ZDS.2*	**1.0 (0.29)**	**0.3 (0.08)**			110.0 (4.49)	165.7 (7.89)	77.2 (6.23)	360.7 (18.88)
*ZDS.5*	**4.0 (0.46)**	**1.4 (0.18)**			89.3 (4.81)	145.1 (8.88)	70.6 (3.40)	317.3 (16.91)
*ZDS.7*	**2.4 (0.40)**	**0.7 (0.14)**			99.5 (3.02)	150.9 (4.68)	70.4 (2.17)	329.6 (9.38)
Anth. Flowers								
*AC++*	0.1 (0.02)	0.1 (0.01)	n.d.		4.6 (0.37)	8.4 (0.71)	82.6 (7.88)	96.3 (8.87)
*ZDS.2*	**17.8 (2.07)**	**5.9 (0.82)**	**11.7 (1.20)**		**1.7 (0.17)**	**6.0 (0.69)**	**34.2 (3.53)**	84.8 (9.11)
*ZDS.5*	**5.9 (0.77)**	**2.4 (0.27)**	**7.08 (0.69)**		**2.3 (0.18)**	7.5 (0.63)	**45.6 (4.62)**	75.9 (6.79)
*ZDS.7*	**14.2 (1.12)**	**5.5 (0.45)**	**10.5 (0.79)**		**2.4 (0.20)**	7.1 (0.57)	**49.1 (3.88)**	94.6 (7.34)
Ripe Fruit								
*AC++*	2.2 (0.21)	1.3 (0.11)	0.3 (0.06)	105.7 (4.15)	7.0 (6.2)	1.7 (0.16)		120.4 (3.89)
*ZDS.2*	**7.6 (1.47)**	**3.4 (0.64)**	**21.7 (4.26)**	**1.1 (0.23)**	**2.9 (0.34)**	**0.5 (0.07)**		**39.6 (6.56)**
*ZDS.5*	**21.6 (2.73)**	**11.4 (1.47)**	**78.9 (12.01)**	**2.0 (0.83)**	8.0 (0.87)	**1.2 (0.23)**		130.5 (15.37)
*ZDS.7*	**18.1 (2.04)**	**9.3 (1.00)**	**71.5 (5.47)**	**0.3 (0.20)**	**4.0 (0.21)**	**0.7 (0.10)**		107.0 (8.95)

Carotenoid contents are presented as μg/g FW (±SEM). Values represent .a mean of a minimum of 3 biological replicates. “n.d.” denotes “not detected”. Bold numbers – indicates significance (*P* < 0.05)

In contrast, photobleaching is prevalent throughout fruit development of *ZDS‐RNAi* lines, with the double‐insert *ZDS.2* line being the most severe (Figure 2b). Accumulation of *all trans*‐lycopene, the characteristic red pigment of ripe tomato fruit, is inhibited throughout ripening of *ZDS‐*RNAi tomato fruit as observed in fruit at the full‐ripe stage (i.e. 7DPB; Figure 2b). Reduced pigmentation was also observed in *ZDS‐RNAi* anthesis flowers compared with wild‐type (Table 1 and Figure S2). Additional phenotypes were observed consistent with reduced synthesis of the carotenoid‐derived hormone ABA in seeds (Figure S4).

Carotenoid profiles in *ZDS‐RNAi* and wild‐type control tissues were assessed using high‐performance liquid chromatography (HPLC). Wild‐type leaves, flowers and ripe fruit accumulated undetectable or low levels of linear carotenoids upstream of ZDS, while *all trans‐*lycopene and/or downstream cyclic carotenoids were abundant (Table 1). CaMV 35S‐driven repression of *ZDS* in leaves, flowers and ripe fruit of the three transgenic lines blocked the pathway to varying degrees, resulting in the accumulation of upstream carotenoids phytoene and phytofluene, while ζ‐carotene isomers only accumulated in anthesis flowers and ripe fruit (Table 1). Downstream photoprotective carotenes (i.e. β‐carotene) and xanthophylls still accumulate to near wild‐type levels (Table 1). It is possible that the modest reduction of *ZDS* mRNA in the leaves as compared to fruit accounts for this observation. In contrast, anthesis flowers and ripe fruit showed a statistically significant decrease in the synthesis of downstream carotenoids (lycopene and β‐carotene), and total xanthophylls compared to wild‐type (Table 1), likely due to the greater inhibition of *ZDS* mRNA accumulation in these tissues.

### *AtZDS* over‐expression reveals an additional bottleneck in fruit ripening‐associated carotenogenesis

To further investigate the relevance of changes in *ZDS* mRNA accumulation in tomato fruit, heterologous over‐expression of *Arabidopsis thaliana ZDS* (*AtZDS*) in tomato driven by the CaMV 35S promoter was employed using the pART27 vector system (Gleave, 1992). As noted above, we deployed heterologous over‐expression rather than use of the tomato *ZDS* gene to reduce potential for co‐suppression of the endogenous gene, a strategy proven useful for manipulation of tomato *PDS* (McQuinn *et al.*, 2017). Nine independent transgenic lines were identified and one, *AtZDS.OE.8*, demonstrated phenotypes similar to *ZDS‐RNAi* lines, suggesting *ZDS* co‐suppression (Figure S5). Of the remaining eight lines, four (*AtZDS.OE.1A*, *AtZDS.OE.4*, *AtZDS.OE.7*, *AtZDS.OE.9)*, each containing a single insert and presenting consistent phenotypic variation, were carried to the T2 generation.

**Figure 3:**
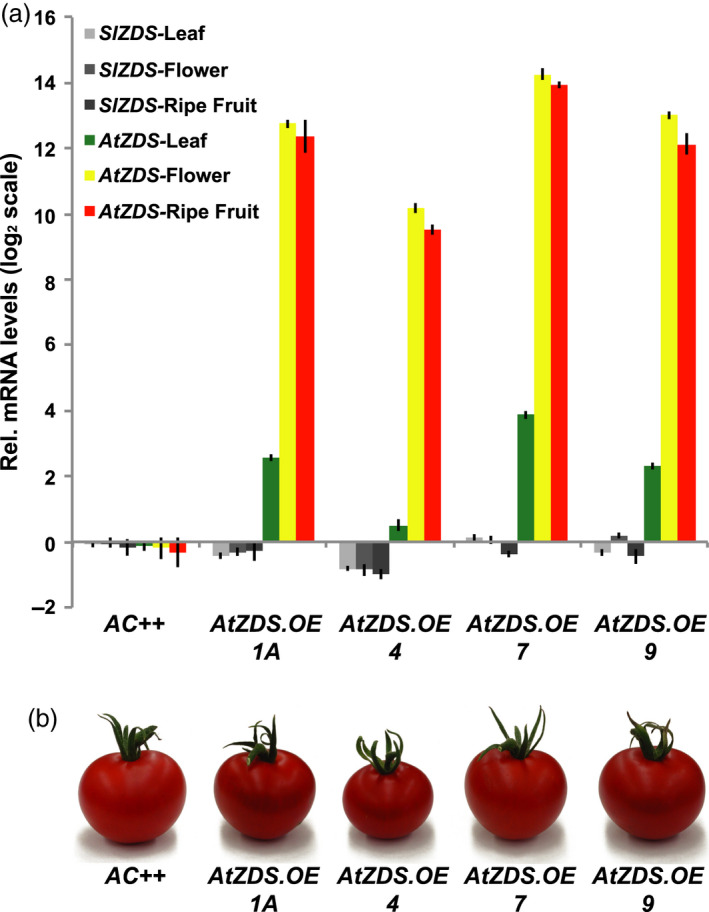
Effectiveness of the AtZDS over‐expression transgene in tomato. (a) Quantitative RT‐PCR comparing tomato and *Arabidopsis* ζ‐carotene desaturase (*SlZDS* and *AtZDS*, respectively) transcript levels in *AtZDS* over‐expression lines relative to the wild‐type (*AC++*). Ripe fruits are 7 days post‐breaker (7DPB) (*n *= 3 and performed in triplicate). (b) Visual phenotypes of chromoplast rich ripe fruit (7DPB) from *AtZDS* over‐expression lines.

The effectiveness of *AtZDS.OE* was determined via qRT‐PCR of the *AtZDS* transgene in young leaves, anthesis flowers and 7DPB fruit from each independent transgenic line (Figure 3). CaMV 35S‐driven expression of *AtZDS* was successful with all tissues analysed displaying substantial transgene mRNA accumulation. The native *Solanum lycopersicum ZDS*, *SlZDS*, is slightly reduced in some of the tissues analysed in the *AtZDS.OE* lines, possibly reflecting a response to the transgene (Figure 3a). Nevertheless, total *ZDS* mRNA was greatly increased in all tested tissues and lines. Young leaves showed the lowest relative increase in *AtZDS* transcript levels ranging from approximately 0.5‐fold to 4‐fold higher when compared to wild‐type (Figure 3a). In chromoplast rich tissues (mature petals and ripe fruits), *AtZDS* transcript levels were elevated to greater than 10 on a log_2_ scale when compared to wild‐type (Figure 3a). Despite successful over‐expression in multiple tissues, visual phenotypes were limited to the ripe fruit at 7DPB (Figure 3b).

Carotenoid profiles of young leaves, flowers and ripe fruits from *AtZDS.OE* lines were investigated via HPLC analysis and compared to carotenoid content in wild‐type control plants. Carotenoid levels remained unchanged in young leaves and flowers in the *AtZDS.OE* lines in agreement with the lack of visual differences (Table 2), suggesting ZDS activity is not limiting. A modest increase in total lycopene content was observed in the 7DPB fruit of the *AtZDS.OE* lines with no significant change in total ζ‐carotene amount (Table 2).

**Table 2:** Carotenoid content in young leaves, anthesis flowers, and ripe fruits of AtZDS.OE lines compared to wildtype (Ailsa Craig) (*n*≥3)

Genotype	Total Phytoene	Total Phytofluene	Total □‐carotene	Total Lycopene	Total □‐carotene	Lutein	Other Xanthophylls	Total Carotenoids
Leaf								
*AC++*	0.07 (0.00)				59.7 (3.82)	95.0 (2.76)	60.9 (1.43)	219.6 (7.68)
*AtZDS.OE.1A*	0.07 (0.01)				59.3 (2.12)	88.6 (1.71)	58.1 (1.65)	209.8 (4.83)
*AtZDS.OE.4*	0.06 (0.00)				53.2 (1.55)	86.2 (2.83)	54.3 (1.51)	198.1 (4.22)
*AtZDS.OE.7*	0.06 (0.01)				63.9 (1.87)	100.0 (4.69)	62.9 (4.26)	231.5 (10.73)
*AtZDS.OE.9*	0.08 (0.01)				60.9 (1.43)	87.7 (2.45)	58.0 (4.16)	211.2 (5.34)
Anth. Flowers								
*AC++*					2.7 (0.17)	6.1 (0.21)	101.5 (4.94)	110.3 (4.94)
*AtZDS.OE.1A*					3.0 (0.11)	6.3 (0.19)	100.3 (4.24)	109.6 (4.18)
*AtZDS.OE.4*					2.3 (0.38)	5.2 (0.14)	104.9 (1.27)	112.3 (1.69)
*AtZDS.OE.7*					2.9 (0.15)	6.2 (0.39)	113.7 (6.78)	122.8 (7.02)
*AtZDS.OE.9*					2.9 (0.14)	5.6 (0.17)	108.9 (1.93)	117.4 (1.97)
Ripe Fruit								
*AC++*	1.7 (0.11)	1.0 (0.06)	0.23 (0.03)	68.4 (2.65)	5.4 (0.14)	1.1 (0.08)		77.9 (2.84)
*AtZDS.OE.1A*	1.9 (0.18)	1.0 (0.10)	0.24 (0.02)	**86.2 (4.02)**	5.9 (0.40)	1.3 (0.06)		**96.5 (4.19)**
*AtZDS.OE.4*	2.1 (0.07)	1.1 (0.03)	0.24 (0.01)	**81.9 (3.59)**	6.4 (0.14)	1.0 (0.05)		**92.8 (3.74)**
*AtZDS.OE.7*	2.0 (0.13)	1.0 (0.06)	0.26 (0.01)	**81.7 (1.50)**	5.1 (0.18)	1.2 (0.06)		**91.2 (1.47)**
*AtZDS.OE.9*	1.7 (0.13)	0.9 (0.06)	0.21 (0.01)	**81.3 (3.65)**	4.9 (0.15)	1.2 (0.07)		**90.2 (3.84)**

Carotenoid contents are presented as □g/g FW (±SEM). Values represent a mean of a minimum of 3 biological replicates. “n.d.” denotes “not detected”. Bold numbers – indicates significance (*P* < 0.05).

### Manipulation of ZDS exposes a dynamic ζ‐carotene isomer profile in ripe tomato fruit

**Figure 4:**
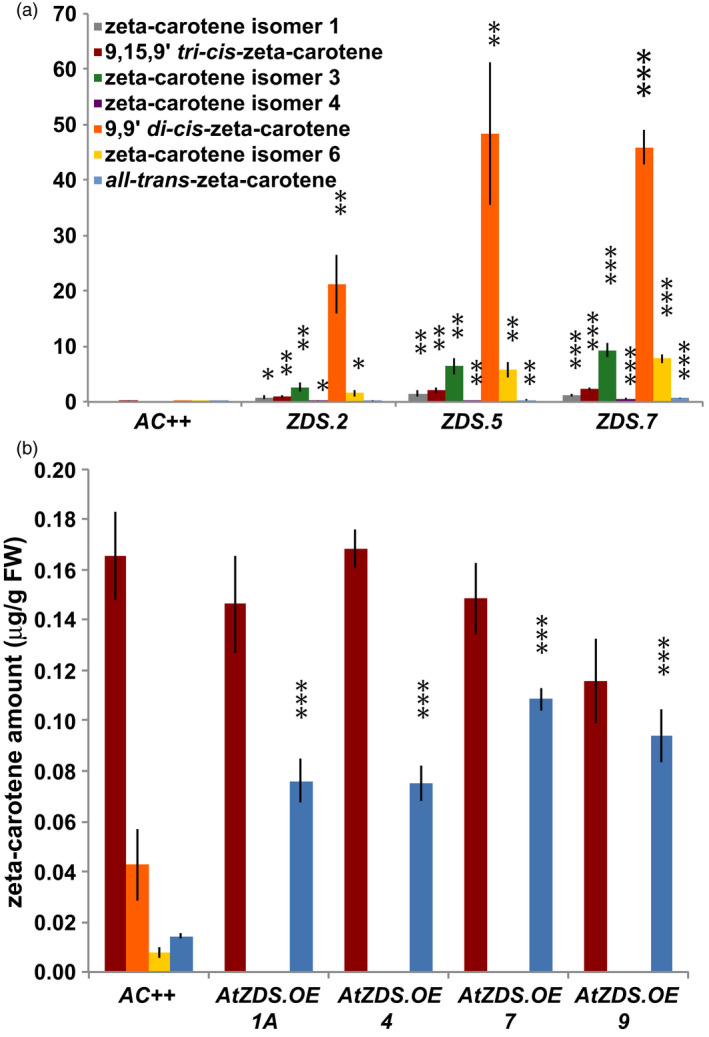
Differential accumulation of ζ‐carotene isomers in ZDS‐RNAi and AtZDS.OE ripe fruit (7DPB). (a) Carotenoid content (µg/g FW) of 7 ζ‐carotene isomers found in ZDS‐RNAi ripe fruit compared to Ailsa Craig wild‐type (*AC++*) (*n* ≥ 3). (b) Carotenoid content (µg/g FW) of 4 ζ‐carotene isomers most commonly found in wild‐type (*AC++*) ripe fruit (7DPB) compared to AtZDS.OE 7DPB fruit (*n* ≥ 5). The legend in A is the same for B. *, *P* < 0.05; **, *P* < 0.01; ***, *P* < 0.001.

While total ζ‐carotene remained unchanged in the *AtZDS.OE* lines, the ζ‐carotene isomer profile was altered greatly in fruit either repressing the endogenous *SlZDS* or over‐expressing *AtZDS* (Figure 4). In *ZDS‐RNAi* fruit, ζ‐carotene isomers 9,15,9′ *tri‐cis‐*ζ‐carotene, 9,9′ *di‐cis‐*ζ‐carotene, 9 *cis‐*ζ‐carotene and *all trans‐*ζ‐carotene were elevated in a manner consistent with that observed in fruit with *ZDS* repressed by VIGS *(*Fantini *et al.*, 2013; Figure 4a). In *AtZDS.OE* fruit, the ZDS substrates, 9,9′ *di‐cis‐*ζ‐carotene and 9 *cis‐*ζ‐carotene were depleted and undetectable while *all trans‐*ζ‐carotene was significantly increased (Figure 4b). This change in the ζ‐carotene isomer profile is consistent with the lack of reduction in the total ζ‐carotene content of AtZDS.OE lines (Table 2).

### Reduced carotenoid content and ABA in *ZDS‐*repressed fruit impacts tomato development

**Figure 5:**
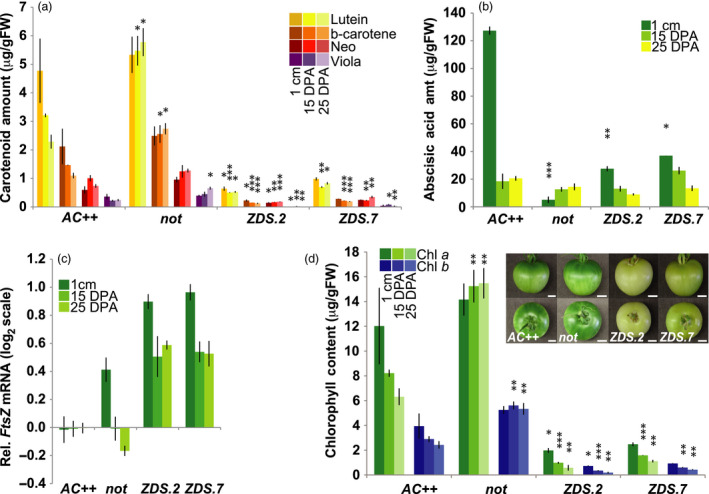
Decreased synthesis of photoprotective carotenoids and ABA in *ZDS‐*repressed tomato fruit. (a) Changes in accumulation of photoprotective carotenoids, lutein; β‐carotene; neoxanthin; and violaxanthin, in ZDS‐RNAi lines and *not* compared to wild‐type (*AC++*) controls through early fruit development. 1 cm, 7–10 days post‐anthesis (DPA); 15 DPA; and 25 DPA (*n* ≥ 3). (b) Decreased ABA synthesis in ZDS‐RNAi lines and *not* compared to wild‐type (*AC++*) through early stages of fruit development (*n* ≥ 3). (c) Elevated *FtsZ* mRNA levels in ZDS‐RNAi lines, *ZDS.2* and *ZDS.7*, and *not* relative to wild‐type (*AC++*) in 3 early stages of fruit development (*n* = 3 and performed in triplicate). (d) Altered chlorophyll *a* and chlorophyll *b* content of ZDS‐RNAi lines and *not* compared to wild‐type (*AC++*) during early stages of fruit development (*n* ≥ 3). Inset picture of photobleached ZDS‐RNAi 25 DPA fruit compared to *not* and *AC++* 25 DPA fruit. Scale bar = 1 cm. *, *P* < 0.05; **, *P* < 0.01; ***, *P* < 0.001.

In contrast to vegetative tissues of ZDS‐RNAi plants, developing fruit displayed clear phenotypes evident of *ZDS* repression and the resulting inhibition of carotenogenesis (Figures 2b and 5). The effects of *ZDS* repression on carotenoids and ABA in developing fruit were determined via HPLC and LC‐MS, respectively, at three stages in early fruit development (10, 15 and 25 DPA). The ABA‐deficient tomato mutant, *notabilis* (*not*), defective in 9‐*cis‐*epoxycarotenoid dioxygenase (SlNCED1) function and minimally characterized with regard to fruit development was included as an additional control (Burbidge *et al.*, 1999).

Levels of *ZDS* mRNA were reduced approximately sixfold in *ZDS.2* and *ZDS.7* developing fruit (Figure S6a). As expected, levels of acyclic carotenoids upstream of ZDS were elevated in *ZDS*‐repressed fruit and undetectable in both wild‐type and the ABA‐deficient *not* mutant (Figure S6b). Levels of photoprotective β‐carotene and xanthophylls were reduced greatly in the *ZDS‐RNAi* lines compared to wild‐type and *not* (Figure 5a)*.* Furthermore, when comparing wild‐type to *not* and the *ZDS‐RNAi* lines, it appears that carotenoid biosynthesis is limited in wild‐type fruit as the cells transition to their predominant expansion phase (1 cm – 15 DPA), while the ABA‐deficient mutant *not, ZDS.2* and *ZDS.7* maintain carotenoid concentration despite increasing cell size (Figure 5a). As suggested by the carotenoid profile in *ZDS‐RNAi*‐developing fruit, *ZDS.2* and *ZDS.7* both have at least 75% reduced ABA accumulation during early stages of fruit development compared to wild‐type and similar to *not* (Rodrigo *et al.*, 2003; Figure 5b).

Reduced levels of ABA in early stages of tomato fruit development result in increased FtsZ‐regulated chloroplast division, thereby elevating plastid number (Galpaz *et al.*, 2008). *FtsZ* transcript levels were measured in *ZDS.2‐* and *ZDS.7*‐developing fruit via qRT‐PCR and compared to wild‐type and *not* fruit. In *not* fruit, a similar increase in *FtsZ* transcripts in early stages of fruit development was observed, consistent with other ABA‐deficient mutants in tomato (i.e. *high‐pigment 3, flacca* and *sitiens*; Figure 5c; Galpaz *et al.*, 2008). *ZDS* repression and resulting ABA deficiency led to the up‐regulation of *FtsZ* expression in *ZDS.2* and *ZDS.7* with *FtsZ* transcript levels twofold higher than the other ABA‐deficient mutants (Figure 5c).

In concert with increased chloroplast division, chlorophyll biosynthetic genes were induced in *not*, *ZDS.2* and *ZDS.7* compared to wild‐type. Chlorophyll *a* oxygenase (*CAO*) expression was elevated significantly in *not* and *ZDS‐RNAi* lines relative to wild‐type (Figure S7a). Expression of protochlorophyllide oxidoreductase (*POR‐B1* and *POR‐B2*), which catalyses the reduction of protochlorophyllide to chlorophyllide, showed a >2‐fold increase in expression in all ABA‐deficient lines with the largest and most prolonged in the *ZDS‐RNAi* lines (Figure S7b). Despite the elevation of chlorophyll biosynthetic gene expression in the *ZDS‐RNAi* lines, *ZDS.2* and *ZDS.7* fruit did not accumulate chlorophyll at levels comparable to wild‐type during development, consistent with reduced photoprotective carotenes and xanthophylls (Figure 2c and 5d) and similar to the effect of reduced *ZDS* on leaves early in development (Figure S2).

**Figure 6:**
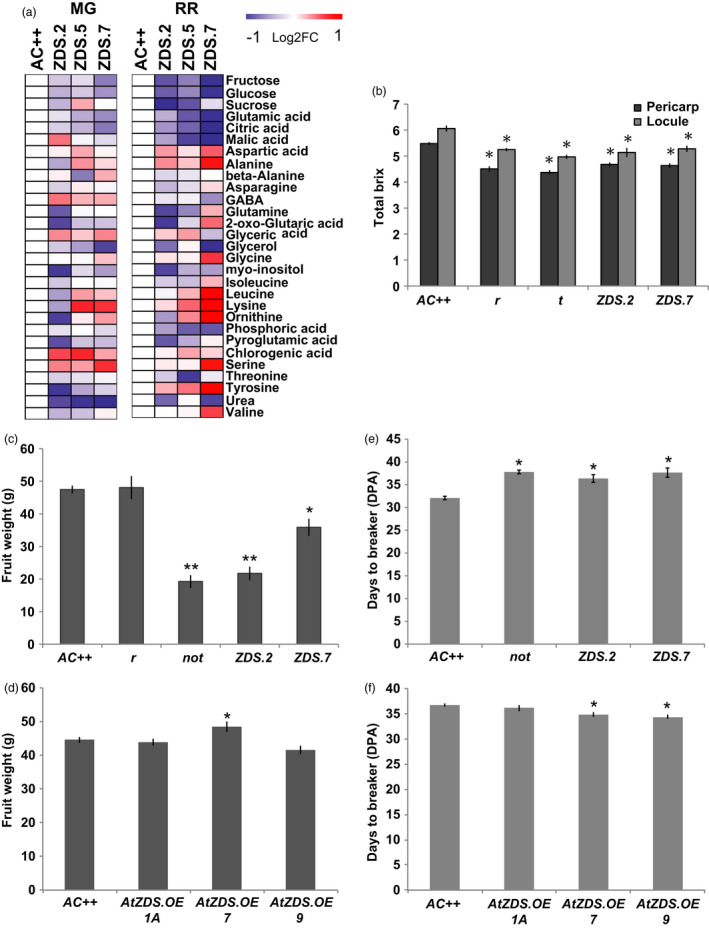
Manipulation of tomato ZDS alters fruit quality and development. (a) Primary metabolite profiling of *AC++* control and ZDS‐RNAi fruit. Intensity of fold change to wild‐type (*AC++*) is visualized by indicating colour (log_2_FC > 2.0 shows red, log_2_FC > −2.0 shows blue). Fruit stages analysed were mature green (MG) and red ripe (RR) (*n* = 6). For metabolite guidelines check list and overview of metabolite list see Table S3 and S4, respectively. (b) Brix^o^ content in 7 DPB fruit pericarp and locule of ZDS‐RNAi lines, *ZDS.2* and *ZDS.7*, compared to other carotenoid mutants, *r/r and tangerine* (*t/t*), and wild‐type (*AC++*) (*n* ≥ 5; *, *P* < 0.05). (c) Decrease in fruit weight of ZDS‐RNAi lines, *ZDS.2*, *ZDS.5* and *ZDS.7,* compared to wild‐type (*AC++*). (d) Fruit weight of AtZDS.OE lines, *AtZDS.OE.1A, 7* and *9* compared to wild‐type (*AC++*). (e) Number of days to reach the initiation of ripening in ABA‐deficient ZDS‐RNAi lines, ZDS.2 and ZDS.7, and *not* compared to wild‐type (*AC++*) (DPA, days post‐anthesis). (f) Number of days post‐anthesis (DPA) to reach the initiation of ripening (breaker) in *AtZDS.OE* lines compared to wild‐type (*AC++*) (*n* ≥ 3; *, *P* < 0.05; **, *P* < 0.01).

We further explored the effects of reduced photosynthetic pigments on attributes of fruit quality (i.e. primary metabolism and brix^o^). A comprehensive assessment of primary metabolism in mature green (MG) and ripe (7DPB) fruit of *ZDS‐RNAi* lines was performed via GC‐MS (Figure 6a). Total soluble solids are negatively influenced in the MG fruit with strong decreases in major brix^o^ components (e.g. fructose, glucose, citric acid; Figure 6a). However, as the fruit ripens the brix^o^ components approach wild‐type levels, though remain lower (Figure 6a). In addition, both stages showed elevated amino acid content consistent with abiotic stress (i.e. photooxidative stress; Obata and Fernie, 2012; Figure 6a). Interestingly, when comparing total brix^o^ levels of 7DPB fruit of *ZDS‐RNAi* lines with same stage fruit of fruit‐specific carotenoid mutants (i.e. *r* and *tangerine*), it is apparent that carotenoids are linked to fruit soluble sugar content during ripening (Figure 6b). It was observed that ripe fruit of *r* and *tangerine*, which lack any visible defect in photoprotection during early fruit development, displays a similar and significant reduction in total brix^o^ levels (Figure 6b).

In addition to influencing chloroplasts and chlorophyll levels during fruit development, reduced ABA levels appear to also impact fruit size and the number of days between pollination and the initiation of ripening (Figure 6c‐f). Post‐breaker fruit were weighed and *ZDS‐RNAi* fruits showed a significant reduction in mass as did the ABA‐deficient *not* mutant (Figure 6c). In *AtZDS.OE* fruit, a significant increase in fruit size was observed in only one of the three lines compared to wild‐type (Figure 6d and Table S2). Ripening initiation was delayed in *ZDS‐RNAi* lines, *ZDS.2* and *ZDS.7*, and the ABA‐deficient mutant, *not,* by approximately 6 days (Figure 6e), consistent with prior observations that exogenous ABA can promote ripening (Zhang *et al.*, 2006). It is noteworthy that this significant ripening impairment has not previously been attributed to tomato ABA‐deficient mutants, presumably because they eventually ripen and the phenotype has not been carefully monitored. Consistently, significant reduction of two days in time to ripening initiation was observed in two of three *AtZDS.OE* lines analysed (Figure 6f and Table S2). Minimal changes in other fruit developmental phenotypes observed in *AtZDS.OE* lines may be indicative of ZDS not being rate‐limiting in photosynthetic tissues, consistent with no detectable levels of ZDS substrates recorded in wild‐type leaves and developing fruit.

**Figure 7:**
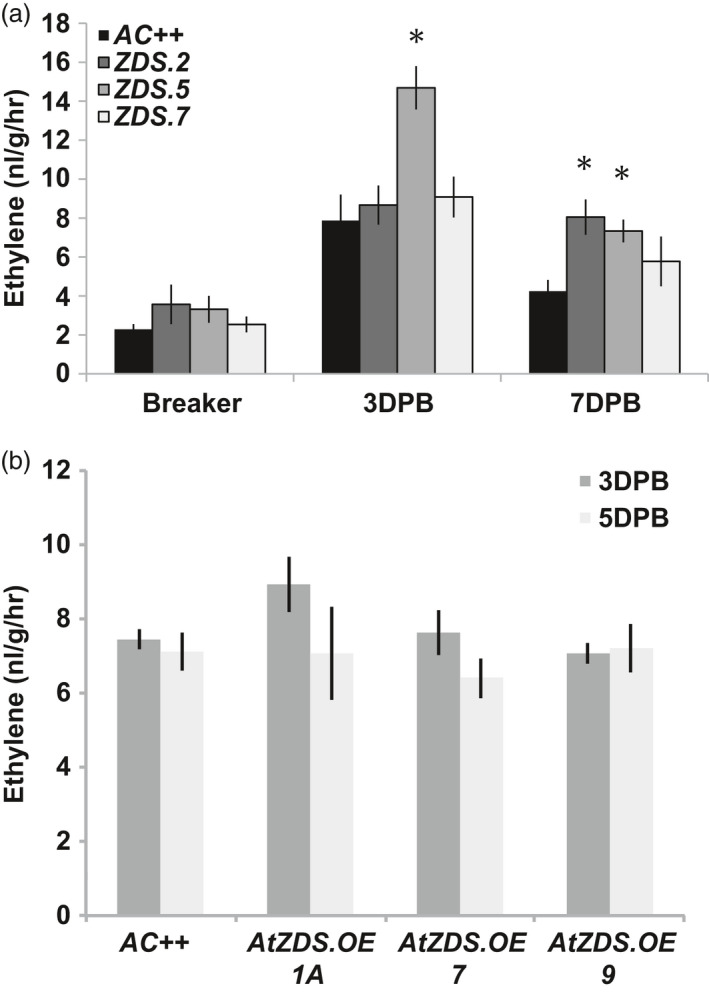
Changes in ethylene production during tomato fruit ripening as a consequence of ZDS manipulation. (a) Ethylene content (ng/g/hr) in breaker, 3DPB and 7DPB fruit of *ZDS‐RNAi* lines, *ZDS.2, ZDS.5* and *ZDS.7,* compared to wild‐type (*AC++*) (*n* ≥ 3; *, *P* < 0.05). (b) Ethylene content (ng/g/hr) in 3DPB and 5DPB fruit of *AtZDS.OE* lines, *AtZDS.OE.1A, AtZDS.OE.7* and *AtZDS.OE.9,* compared to wild‐type (*AC++*) (*n *> 5).

ABA and ethylene have been demonstrated to have an antagonistic relationship during plant development including leaf epinasty and adventitious root growth as observed in the ABA‐deficient tomato *not* mutation, and during fruit ripening in the ABA‐deficient tomato *hp3* mutant (Galpaz *et al.*, 2008; Thompson *et al.*, 2004). During fruit ripening of *ZDS‐RNAi* lines, ripening fruit displayed elevated or prolonged ethylene production (Figure 7a). However, no complementary effect was observed in *AtZDS.OE* ripening fruit where ethylene levels remain comparable to wild‐type (Figure 7b).

## Discussion

Current understanding of ripening‐associated carotenoid biosynthesis has been largely established through the cloning and functional characterization of genes underlying natural fruit‐specific carotenoid mutants in tomato (Fray and Grierson, 1993; Galpaz *et al.*, 2006; Isaacson *et al.*, 2002; Ronen *et al.*, 2000). Both desaturase genes (*PDS* and *ZDS*) remain undefined by tomato mutations, likely due to their single copy gene status and anticipated deleterious effects on photosynthesis impeding survival and consistent with *Arabidopsis* and *Zea mays ZDS* mutants (Dong *et al.*, 2007; Matthews *et al.*, 2003). We implemented transgenic repression of tomato *ZDS* and heterologous over‐expression of *AtZDS* to provide insight into how the manipulation of ZDS and carotenogenesis may alter tomato fruit development and quality.

### The single copy ZDS is up‐regulated at ripening initiation by NAC‐NOR, yet represents a second bottleneck in ripening‐associated carotenogenesis

While most steps of the carotenoid biosynthetic pathway are associated with small gene families including members expressed specifically in chloroplast‐ or chromoplast‐rich tissues, we confirm that ZDS is a single copy gene in tomato crucial for carotenoid biosynthesis throughout plant development. Ripe fruit accumulate elevated levels of carotenoids as attractants for seed dispersing frugivores and are largely dependent upon transcriptional induction of *ZDS* (Figure 1a). *ZDS* transcript levels are dynamic and tightly regulated in developing and maturing fruit. Prior work on tomato ripening mutants (i.e. *rin, nor, Nr, Gr*) has demonstrated that key steps in the carotenoid pathway are targets of enhanced transcription as fruits ripen to increase flux towards lycopene (Alba *et al.*, 2005; Barry *et al.*, 2005; Vrebalov *et al.*, 2009; Vrebalov *et al.*, 2002). *PSY1* is well described as the rate‐limiting step in fruit carotenogenesis and positively up‐regulated by ethylene perception and signalling and the RIN transcription factor (reviewed in Gapper *et al.*, 2013). We demonstrate that *ZDS* transcription is controlled via a complex array of ripening regulatory components in a manner similar to *PSY1*, including ethylene signal transmission and two ripening transcription factors, the MADS‐box transcription factor RIN and NAC domain transcription factor NAC‐NOR (Gapper *et al.*, 2013; Giovannoni *et al.*, 2004; Wang *et al.*, 2019; Figure 1c).

*ZDS* transcripts were significantly reduced in ripe fruit of *Nr* and *Gr,* both dominant mutations in an ethylene receptor and a receptor‐associated protein, respectively, conferring ethylene insensitivity suggesting ethylene signalling is critical for *ZDS* transcriptional regulation via a potential interaction with ethylene response transcription factors or ERF’s (Adams‐Phillips, Barry & Giovannoni,^2004^ and Gapper *et al.*, 2013). Similar and stronger repression of *ZDS* was observed in 7DPB fruit of *rin* and *nor,* respectively (Figure 1c). At first glance, this repression of *ZDS* may reflect an indirect regulation through RIN’s and NAC‐NOR’s influence on ethylene (Adams‐Phillips *et al.* 2004; Ito *et al.*, 2017; Wang *et al.*, 2019). On the contrary, upon mining available databases of RIN and NAC‐NOR ChIPSeq data (ted.bti.cornell.edu and www.epigenome.cuhk.edu.hk/encode.html), both transcription factors bind to the promoter region immediately upstream of the transcriptional start site, suggesting a role in the direct regulation of *ZDS* (Vrebalov *et al.*, 2002, Wang *et al.*, 2019 and reviewed in Giovannoni *et al.*, 2017). Herein, we demonstrate that ZDS represents an additional carotenoid biosynthetic gene heavily regulated at the onset of and throughout tomato fruit ripening by ripening transcription factors and ethylene signalling either separately or in combination.

Consistent with *ZDS* expression in the wild‐type cultivar (Figure 1a), repression of *ZDS* via the CaMV 35S promoter inhibits carotenoid biosynthesis downstream of 9,9′ *di‐cis*‐ζ‐carotene significantly in flowers and fruits (Table 1). Further, while the modest repression of *ZDS* in young leaves allows small quantities of the upstream acyclic carotenoids to accumulate, no significant changes are observed in downstream photoprotective carotenes and xanthophylls and phytohormone (i.e. ABA and strigolactone) precursors after acclimation to normal light conditions (Table 1). That said, a temporary susceptibility to highlight was observed upon transition from low light to normal light conditions, which was overcome quickly as the plant acclimated despite constant repression of *ZDS* expression (Figure S2 and Table 1), similar to the effects of the weak *ZDS* allele encoded by the Arabidopsis *spc1* mutation (Dong *et al.*, 2007). We surmise that it may be possible that weak repression results in low levels of downstream cyclic carotenoids, which then undergo non‐enzymatic oxidative cleavage by singlet oxygen due to increased susceptibility to photoinhibition. The resulting apocarotenoid signal (i.e. β‐cyclocitral) may provide a means of acclimation to the increased light stress as previously reported (Ramel *et al.*, 2012).

As the focus of this study was to investigate the impacts of *ZDS* manipulation on ripening‐associated carotenogenesis and tomato fruit development, *ZDS‐RNAi* lines with stronger repression and albino phenotypes (i.e. *ZDS.1* and *ZDS.8*) were excluded from this study (Figure S2) to facilitate viability through fruit set. Indeed, this phenotypic severity suggests greater ZDS reduction is highly detrimental to plant development and survival. Further assessment of these more severe genotypes may provide insight into the role of the previously described higher plant ζ‐carotene‐derived apocarotenoid signal (Avendano‐Vasquez *et al.,* 2014). Consistent with a single *ZDS* gene, additional phenotypes were observed in the weaker repression lines with impaired synthesis of the carotenoid‐derived hormone ABA in seeds causing precocious seed germination like that observed in the maize *ZDS* mutant, *vp9* (Matthews *et al.*, 2003; Figure S4).

Over‐expression of *AtZDS* in tomato generates a modest increase in downstream *all trans*‐lycopene of the ripening fruit (Table 2). Concurrently, there is a total depletion of the ZDS substrate 9,9′ *di‐cis‐ζ*‐carotene, which otherwise persists at low levels in wild‐type tomato fruit (Figure 4b). The steps catalysed by ZDS thus represent a minor limitation to maximal potential activity of the wild‐type biosynthetic pathway. ZDS may be an effective target for genetic manipulation when its substrate is elevated, such as in the case of the recently described tomato *AtPDS* over‐expression lines (McQuinn *et al.*, 2017).

### Carotenoids and ABA influence fruit development and ripening

Abscisic acid is one of two carotenoid‐derived hormones (the other being strigolactones), characterized to date. ABA is synthesized throughout the plant aiding in protection against abiotic stresses (i.e. drought, salt and cold) in addition to participating in the regulation of plant development (e.g. seed germination and fruit ripening; reviewed in Nambara and Marion‐Poll, 2005). As fruit of multiple plant species develop and mature, ABA undergoes dynamic shifts in its synthesis and catabolism. The first is often during early development and fruit expansion followed by a sharp decrease before the onset of ripening. In tomato and other species such as persimmon, there is an induction of ABA prior to ripening initiation and at least in tomato, capable of inducing the ripening hormone ethylene (Leng *et al.*, 2014). In other fruit, ABA is increased in concert with later maturation, ripening and/or senescence (Cotton: Davis and Addicott, 1972; Persimmons: Zhao *et al.*, 2012; Peach: Soto *et al.*, 2013; Avocado: Chernys and Zeevaart, 2000). Strawberry fruit are highly dependent on ABA for complete ripening (Jia *et al.*, 2011).

While more recent studies indicate that ABA is important in tomato ripening based on its timing of accumulation and ability to initiate ripening when applied exogenously (Mou *et al.*, 2016; Zhang *et al.*, 2009), its precise role in tomato ripening remains unclear. Tomato ABA‐deficient mutants provide an opportunity to explore the significance of ABA on ripening. Galpaz *et al. *(2008) demonstrated ABA’s negative regulation of tomato fruit chloroplast division at the transition from cell division to cell expansion through characterization of the ABA‐deficient mutant *high‐pigment 3* (*hp3*). Reduced ABA in developing fruit of our *ZDS‐RNAi* lines resulted in increased *FtsZ* expression mimicking the *hp3* effect and supporting increased plastid division and associated elevated plastid number as resulting from loss of ABA early in fruit development (Galpaz *et al.*, 2008; Figure 5b and c). The chlorophyll biosynthetic genes, *POR B* and *CAO*, are elevated consistent with increased plastid numbers, yet decreased chlorophylls were observed, in contrast to the ABA‐deficient *not* mutant (Figure S7 and Figure 5d). Reduced chlorophyll in *ZDS* repression lines may reflect a reduction in photoprotective carotenes and xanthophylls (Figure 5a). Interestingly, changes in chlorophyll biosynthetic gene expression differ substantially from that observed in the *Arabidopsis spontaneous cell death1* (*spc1*) mutants, suggesting that developing fruit may possess a different retrograde signalling network in response to dysfunctional chloroplasts or chlorophyll deficiency.

ABA content is strongly linked to mature green fruit size, the stage preceding ripening initiation. Galpaz *et al. *(2008) studied the tomato ABA‐deficient mutants, *flacca, sitiens and hp3,* and observed mature fruit weight was approximately 75% of control fruit for all three mutants. 30%–40% reductions in fruit weight were also observed for transgenic tomatoes with repressed *NCED1* expression (Sun *et al.*, 2012). The 30%–50% reduction in fruit weight of the *ZDS‐RNAi* lines is consistent with these observations and their resulting decrease in ABA (Figure 6c). Taken together, these results demonstrate that ABA plays an important role in fruit size determination and levels of the hormone are influenced by changes in upstream carotenoid synthesis enzymes including ZDS.

Climacteric fruit such as tomato produce a characteristic ethylene burst at the onset of ripening, which is necessary for maturation to proceed (Gapper *et al.*, 2013; Giovannoni *et al.*, 2017). The synthesis and perception of ABA during fruit development has been proposed as an early ripening regulator upstream of ethylene (Zhang *et al.*, 2009). Inhibition of ABA biosynthesis in developing tomato fruit via a carotenoid synthesis inhibitor (NDGA) delayed initiation of ripening for 3 days (Zhang *et al.*, 2009). The ABA‐deficient *pinalate* mutant in *Citrus sinensis* displays a similar effect in that de‐greening is prolonged (Rodrigo *et al.*, 2003). The 7‐day delay of ripening initiation in *ZDS‐RNAi* tomato fruit further supports a strong relationship between ABA and fruit ripening (Figure 6e). While the ABA‐deficient mutant *notabilis* was previously shown to inhibit fruit expansion, we show here that it is also ripening delayed (Figure 6e), while fruit from two out of the three *AtZDS* over‐expression lines tested ripen on average 2 days earlier than wild‐type fruit (Figure 6f and Table S2). Further, altered plastid levels are known to associate with fruit sugar/TSS content (Nguyen *et al.*, 2014) but the effect of altered carotenoid flux on ABA and downstream signalling molecules may indicate a more direct role of carotenoid metabolism on ripening control. Indeed, other carotenoid impaired mutants such as *lutescent2* (*l2*) that fail to accumulate carotenoids at the onset of ripening due to impaired plastid accumulation are significantly ripening delayed (Barry *et al.*, 2012) further supporting a role of carotenoids in coordination of ripening process and beyond their effects on ripe fruit pigmentation and associated appearance and nutrient quality.

In short, we confirm that ABA influences fruit development and ripening at many junctures. Initially as a mediator of fleshy fruit expansion and later as a component of the complex set of genetic and hormonal interactions that contribute to ripening induction. As the fruit matures, ABA influences ethylene synthesis via its well‐documented antagonistic relationship (Cheng *et al.*, 2009). In *ZDS‐RNAi* repression lines, ethylene synthesis is maintained at a higher level for a longer period consistent with observations from the ABA‐deficient tomato *hp3* mutant (Galpaz *et al.*, 2008; Figure 7a). Together, these results further suggest that in contrast to observations in leaves of *Arabidopsis ZDS* mutants (*chloroplast biogenesis 5* (*clb5*) and *spc1*), the overaccumulation of upstream carotenoids (i.e. phytofluene and ζ‐carotene) does not produce any identifiable alterations in tomato fruit development that differ from the expected phenotypes attributed to ABA and carotenoid deficiency.

## Experimental procedures

### Plant materials and growth conditions

Wild‐type (*Solanum lycopersicum* cv Ailsa Craig, LA2838A) and homozygous *notabilis* (LA3614); *Never ripe* (Barry *et al.*, 2005); *Green ripe* (LA2435); *ripening‐inhibitor* (LA3754); and *non‐ripening* (LA3770) mutant seed were obtained from the Tomato Genetics Resource Center, UC Davis (http://tgrc.ucdavis.edu/). Plants were grown in greenhouses at the Guterman Bioclimate Laboratory and Greenhouse Complex, Cornell University, Ithaca, NY. All plants were grown under low light conditions consisting of 12‐h day/night and under a mesh shade cloth for the first month after which plants were transplanted, removed from shade and grown under long day conditions (16‐hr days and 8‐h nights). ZDS‐RNAi transgenic lines were carried on to the T1 generation where heterozygotes were selected for further analysis. Analysis of the AtZDS over‐expression lines was carried out to the T2 generation.

Young leaf tissue was collected off the 4th, 5th and 6th leaves from the meristem of two‐month‐old plants. Whole petals and anthers were harvested from flowers at anthesis/pollination (the day a flower fully opened). Developing fruit was staged relative to pollination by tagging flowers at anthesis and collecting fruit at specific days post‐anthesis (i.e. 10, 15, 25 and 30DPA/MG). Ripening stages were determined as days post‐breaker where breaker is the initiation of overt ripening – initial colour change at the blossom end of the fruit. Zygosity and copy number of transgenic lines were determined in the T1 generation and confirmed in the T2 generation.

### RNAi and over‐expression constructs for plant transformation

The ZDS‐RNAi construct was made in the pHELLSGATE 2 vector (provided by Peter Waterhouse, University of Sydney, Australia). The ZDS cDNA sequence used in construct development spans 285bp of the 3′UTR starting from the most 5′ bp of the stop codon. PCR amplification of the RNAi fragments was accomplished using the FastStart High‐fidelity PCR system (Cat No. 04‐738‐292‐001, Roche Applied Sciences, IN) using the EST clone TUS‐69‐K8 with primers ZDSRNAi‐for and ZDSRNAi‐rev containing recombination sequence specified in the Gateway BP Clonase kit (Cat No. 11789‐020, Invitrogen, CA; Table S3). The ZDS‐RNAi fragment was Gel purified (Cat No. 28706, Qiagen, MD) and cloned into pHELLSGATE 2 according to BP Clonase kit instructions. The resulting construct was sequence verified and transformed into *S. lycopersicum* cv Ailsa Craig by *Agrobacterium tumafaciens* (strain LBA‐4404) as described in Van Eck *et al. *(2006).

The AtZDS over‐expression (AtZDS.OE) construct was generated as described previously (Gleave, 1992; McQuinn *et al.*, 2017). RNA from Arabidopsis thaliana (accession Columbia‐0) leaf tissue was converted to cDNA via iScript™ cDNA synthesis kit (Cat. No. 170‐8891, Bio‐Rad, CA). Resulting cDNA was used to amplify the full length AtZDS ORF via FastStart High‐fidelity PCR system (Cat. No. 04‐738‐292‐001, Roche Applied Sciences, IN) with AtZDS‐OE‐KpnI.for and AtZDS‐OE‐XbaI.rev primers (Table S3). The resulting AtZDS.OE construct was sequence verified and transformed into *S. lycopersicum* cv. Ailsa Craig by *Agrobacterium tumafaciens* (strain LBA‐4404) as described in Van Eck *et al. *(2006).

### DNA isolation and zygosity/copy number analysis

Genomic DNA was isolated from fresh meristematic leaf tissue as previously described Barry *et al. *(2005). Verification of insertion events in the ZDS‐RNAi T0 plants was carried out via PCR using primers specific for the 35S promoter in the pHELLSGATE 2 vector (Table S3). Insertion of *AtZDS* was validated using internal primers within the 35S promoter and the *AtZDS* ORF (Table S3). Zygosity and copy number was determined in the T1 generation via quantitative PCR relative to the single copy polygalacturonase 2a gene (PG2a, GenBank accession No. X04583) as previously described (McQuinn *et al.*, 2017).

### RNA isolation and quantitative RT‐PCR analysis

Total RNA was isolated using a modified protocol from the RNeasy Minikit (Cat. No 74106, Qiagen Sciences, MD) as described in McQuinn *et al. *(2017). Quantitative real‐time PCR was performed using the Power SYBR^®^ Green RNA‐to‐C_T_™ 1‐Step Kit (Cat No. 4309169, Applied Biosystems, NJ) in a 5 µL reaction volume (2.5 µL 2X Master Mix; 1 µm forward and reverse primers; 1 µL of total RNA; 0.46 µL DEPC‐treated water; 0.04 µL RT enzyme mix). All tissue samples were represented by a minimum of 3 biological replicates, each with triplicate technical replicas. Gene‐specific primers were checked for efficiency using wild‐type or reference RNA (for primer sequence, see Table S3). To be able to apply the standard curve method described in User Bulletin #2 (Applied Biosystems, 1997), a standard curve was included on each plate for the specific gene being analysed using wild‐type or reference RNA (serial dilutions: 50 ng; 5 ng; 0.5 ng; 0.05 ng; 0.005 ng) in triplicate. For each gene analysis, template‐free and negative‐RT controls were included. Real‐time PCRs were carried out using an ABI PRISM™ 7900HT Sequence Detection System (Applied Biosystems, CA) under the following reaction conditions: reverse transcription at 48°C for 30 min; enzyme activation at 95°C for 10 min; followed by 40 cycles of 95°C for 15 s and 60°C for 1 min. A dissociation curve was added at the end of the run for verification of primer specificity.

ABI PRISM™ SDS version 2.3 software (Applied Biosystems, California) was used to determine gene‐specific threshold cycles (C_T_) using the endogenous reference (18S rRNA) for every sample. C_T_’s were extracted, and the standard curve method (Applied Biosystems, 1997) was applied to calculate relative mRNA levels in comparison to the wild‐type control or the reference sample (equal volume and concentration of RNA from each tissue combined).

### Carotenoid and chlorophyll extraction and analysis

Carotenoids and chlorophylls were extracted from 200 mg of leaf tissue and frozen tomato pericarp, while carotenoid and xanthophyll contents of anthesis flowers were extracted from replicates of petals and anthers of a single flower. Tissue prep, pigment extraction and analysis were carried out using a modified protocols from Alba *et al. *(2005) as described previously (McQuinn *et al.*, 2017). All solvents used were HPLC grade.

### Brix^o^ (TSS) content quantification

Brix^o^ content was measured from pericarp juice and pulp separately in freshly harvested ripe fruit with a digital refractometer (Artisan™ HR200 Digital Refractometer; APT Instruments; IL).

### Ethylene measurements

Ethylene was measured from fruits by sealing the fruit in airtight jars for 3 h at 22°C, after which a 1ml aliquot of headspace gas for each fruit was injected on to an Agilent 6850 Network GC System equipped with a flame ionizing detector (Agilent technologies; CA). Samples were compared to standards of known concentration and normalized for fruit mass.

### ABA extraction and quantification

Abscisic acid (ABA) levels were extracted from fruit via a modified extraction method described in Thaler *et al. *(2010) designed for the extraction and quantification of ABA and 3 additional plant hormones (i.e. salicylic acid (SA), jasmonic acid (JA) and auxin/indole‐3‐acetic acid (IAA)). Modification was addition of 100 µL of internal standard containing 0.8 ng/µL each of D_6_‐ABA, D_4_‐SA, D_5_‐JA and D_5_‐IAA to each sample prior to homogenization. ABA levels were quantified upon application of a 10ml aliquot of each sample on a triple‐quadrupole LC‐MS/MS (Thermo Scientific®, Waltham, MA, USA) equipped with a C_18_ reverse‐phase HPLC column (Gemini‐NX, 3 mm, 150 × 2.00 mm, Phenomenex, Torrance, CA) using the method described in Thaler *et al. *(2010). ABA was analysed by negative electrospray ionization (spray voltage: 3.5 kV; sheath gas: 15; auxiliary gas: 15; capillary temperature: 350°C) with a collision‐induced dissociation (CID) energy of 13V (argon CID gas pressure 1.3 mTorr [1.3 micron Hg]) and selected reaction monitoring (SRM) of compound‐specific [parent → product ion] transitions: ABA[263 m*/z* → 153 m/z] D_6_‐ABA as described in Rasmann *et al. *(2012).

### Primary Metabolite analysis using GC‐TOF‐MS

Metabolite analysis of primary metabolites by GC‐MS was performed as described by Lisec *et al. *(2006) and optimized for tomato fruit pericarp as described in Rohrmann *et al.* (2011). Chromatograms and mass spectra were evaluated with Chroma TOF 1.0 (Leco) and TagFinder 4.0 software (Luedemann *et al.*, 2008). Further supporting information regarding  the metabolite analysis is available in Tables S4 and S5. 

### Statistical analysis

Statistical analysis and comparison of mean values (i.e. carotenoid, xanthophyll and chlorophyll amounts, ABA, brix^o^, fruit size, days to ripening and ethylene) from each mutant genotype to the control genotype were performed using the Student’s *t*‐test.

## Conflicts of Interest

All authors declare there is no conflict of interest.

## Authors Contributions

RPM and JJG conceived the research project and designed the experiments. RPM, NEG, ASG, SZ and TT carried out the experiments. ZF, ARF and JJG supervised the experiments. RPM wrote the article, under the supervision and complementary writing of JJG. JJG agrees to serve as the author responsible for contact and ensures communication.

## Supporting information


**Figure S1.** The carotenoid biosynthetic pathway noting *S. lycopersicum* mutations*.*
**Figure S2.** Additional phenotypes in ZDS‐RNAi lines.
**Figure S3.** ZDS repression construct homozygosity results in severe deleterious phenotypes.
**Figure S4.** Evidence of reduced ABA accumulation in ZDS‐RNAi seeds.
**Figure S5.** T_0_ generation ripe fruit images reveals co‐suppression in line *AtZDS.OE.8*
**Figure S6.** Transgene efficiency in ZDS‐RNAi developing fruit.
**Figure S7.** Low ABA induced chlorophyll biosynthesis during early stages of fruit development in ZDS‐RNAi lines.

**Table S1.** Carotenoid gene families with chloroplast and chromoplast specificity.
**Table S2.** Fruit weight (g) and days to breaker of AtZDS.OE lines compared to AC++ (±SE).
**Table S3.** Primer list.

**Table S4.** Metabolite Reporting Guidelines (Checklist table).
**Table S5.** Overview of the metabolite reporting list.
