# Supplementary material for: Manipulation of ZDS in tomato exposes carotenoid‐ and ABA‐specific effects on fruit development and ripening
Source: Plant Biotechnol J. 2020 Apr 20;18(11):2210–24. doi: 10.1111/pbi.13377 (PMC7589306; doi:10.1111/pbi.13377)
Supplement: Supplementary file 1 — Figure S1. The carotenoid biosynthetic pathway noting S. lycopersicum mutations. Figure S2. Additional phenotypes in ZDS‐RNAi lines. Figure S3. ZDS repression construct homozygosity results in severe deleterious phenotypes. Figure S4. Evidence of reduced ABA accumulation in ZDS‐RNAi seeds. Figure S5. T0 generation ripe fruit images reveals co‐suppression in line AtZDS.OE.8 Figure S6. Transgene efficiency in ZDS‐RNAi developing fruit. Figure S7. Low ABA induced chlorophyll biosynthesis during early stages of fruit development in ZDS‐RNAi lines. [file PBI-18-2210-s001.docx]

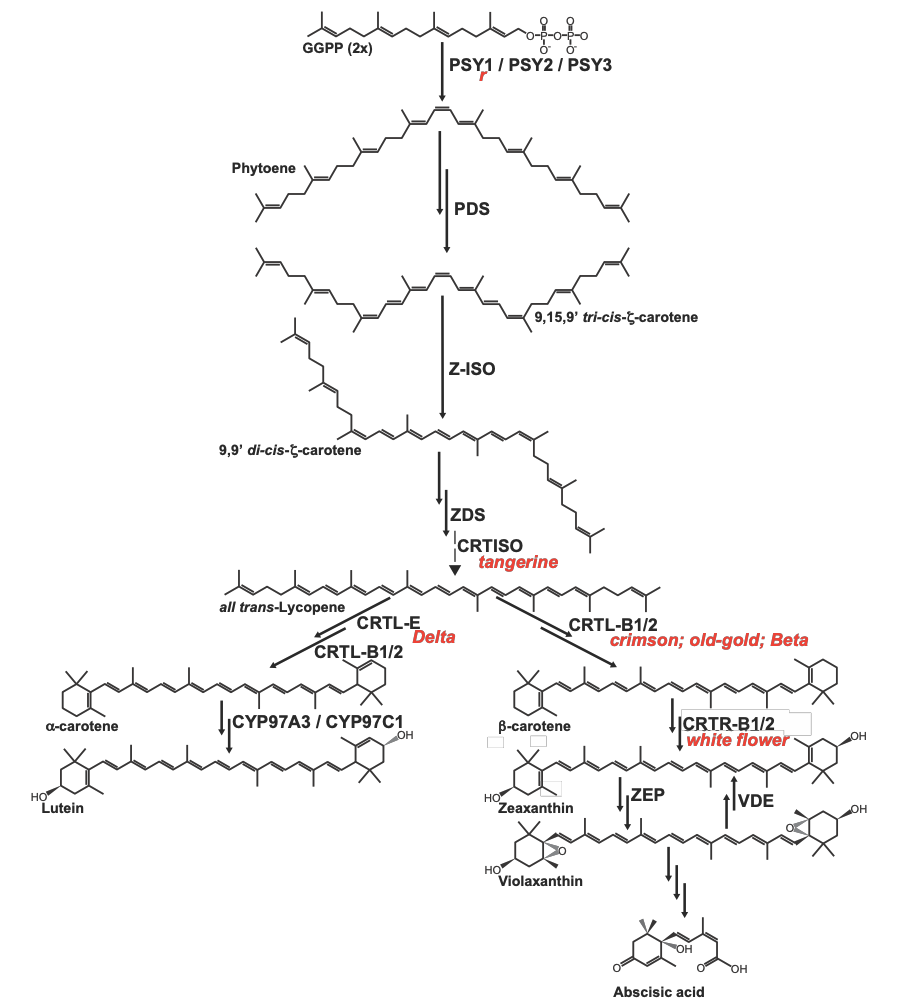
**Figure S1. The carotenoid biosynthetic pathway noting *S. lycopersicum* mutations*.*** Metabolites: GGPP, geranlgerany diphosphate; Enzymes: PSY1, phytoene synthase (chromoplastic); PSY2, phytoene synthase (chloroplastic); PSY3, phytoene synthase 3; PDS, phytoene desaturase; Z-ISO, ζ-carotene isomerase; ZDS, ζ-carotene desaturase; CRTISO, carotene isomerase; CRTL-B1/2, lycopene β-cyclase (chloroplastic/chromoplastic); CRTL-E, lycopene ε-cyclase; CRTR-B1/2, carotene β-hydroxylase (chloroplastic/chromoplastic); CYP97A3, carotene β-hydroxylase; CYP97C1, carotene ε-hydroxylase; ZEP, zeaxanthin epoxidase; VDE, violaxanthin de-epoxidase.


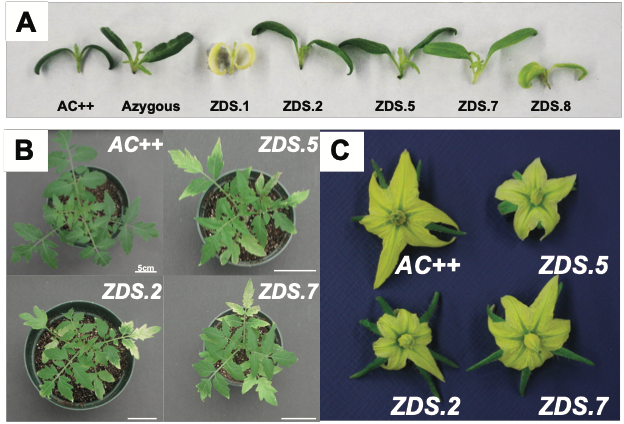


**Figure S2. Additional phenotypes in ZDS-RNAi lines.** A, ZDS-RNAi resulted in varying degrees of severity of photobleaching in vegetative tissues. B, Lines with minimal deleterious phenotypes in vegetative tissues were selected for further fruit specific analyses. C, ZDS-RNAi plants develop viable flowers with reduced pigmentation.


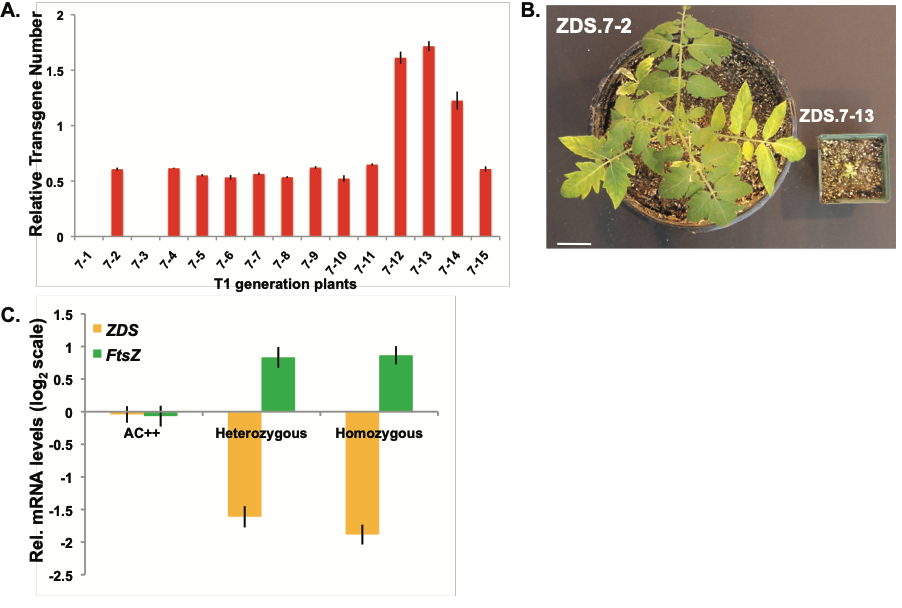


**Figure S3. ZDS repression construct homozygosity results in severe deleterious phenotypes.** A, ZDS RNAi transgene number and zygosity relative to the native single copy *SlPG2a*. Ratio close to 1:2:1 for a segregating population with a single transgene insertion. Lines with a value around 0.5 represent heterozygotes, while lines 12,13, and 14, represent the homozygotes. Lines 1 and 3 represent Azygous lines. B, Comparison of heterozygous and homozygous plants at the same age and under the same growth conditions. C, Comparison of repressed ZDS transcript levels in heterozygous and homozygous plants (i.e. 7-2 and 7-13, respectively). *FtsZ* is included to show that there are similar changes in the expression of ZDS associated genes.


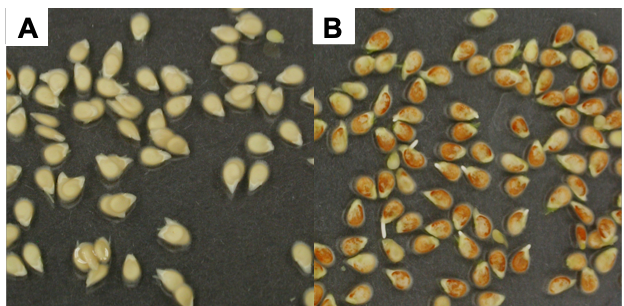


**Figure S4. Evidence of reduced ABA accumulation in ZDS-RNAi seeds.** A, Seed extracted from wild-type (AC++) breaker fruit. B, Seed extracted from ZDS-RNAi 2 breaker fruit. Shows precocious seed germination and seed discoloration when compared to wild-type.

**
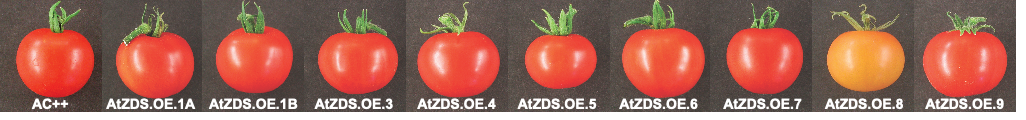
**

**Figure S5. T_0_ generation ripe fruit images reveals co-suppression in line *AtZDS.OE.8.***

**Figure S6. Transgene efficiency in ZDS-RNAi developing fruit.** A, ZDS repression in developing fruit of ZDS-RNAi lines (ZDS.2 and ZDS.7) relative to wild-type (AC++). Stages of development are 1cm, approximately 7 days post anthesis (DPA); and 15 and 25 DPA (*n*=3 each in triplicate) B, Carotenoid content of ZDS.2 and ZDS.7 during 3 stages of early fruit development compared to wild-type (AC++) and ABA deficient mutant (*not*). Specific to carotenoids upstream of ZDS (Phytoene, Phytofluene, and ζ-carotene) (*n*>3).

**Figure S7. Low ABA induced chlorophyll biosynthesis during early stages of fruit development in ZDS-RNAi lines.** A, Chlorophyll a oxyenase mRNA levels in ZDS-RNAi lines and *not* relative to wild-type (AC++) in early fruit development. B, Protochlorophyllide oxidoreductase expression in ZDS.2, ZDS.7, and *not*, relative to wild-type (AC++) in early fruit development (*n*=3 each in triplicate).
