# Supplementary material for: Manipulation of ZDS in tomato exposes carotenoid‐ and ABA‐specific effects on fruit development and ripening
Source: Plant Biotechnol J. 2020 Apr 20;18(11):2210–24. doi: 10.1111/pbi.13377 (PMC7589306; doi:10.1111/pbi.13377)
Supplement: Supplementary file 2 — Table S1. Carotenoid gene families with chloroplast and chromoplast specificity. Table S2. Fruit weight (g) and days to breaker of AtZDS.OE lines compared to AC++ (±SE). Table S3. Primer list. [file PBI-18-2210-s003.docx]

| **Table S1. Carotenoid gene families with chloroplast and chromoplast specificity** | | | | |
| --- | --- | --- | --- | --- |
| Enzymatic Step | No. of genes | Chloroplast | Chromoplast | Citation |
| GGPP Synthase | 2 | GGPS1^c^ | GGPS2^c^ |  |
| Phytoene Synthase | 3 | PSY2 | PSY1  (*r*/*r*) | Fraser *et al.* (1999); Fray and Grierson (1993) |
| Phytoene desaturase | 1 | PDS^a,b,c^ | PDS^a,b,c^ | Qin *et al.* (2007); Matthews *et al.* (2003) |
| ζ-carotene isomerase | 1 | * | ZISO | Li and Murillo (2007) |
| ζ-carotene desaturase | 1 | ZDS^a,b,c^ | ZDS^a,b,c^ | Dong *et al*. (2007); Matthews *et al.* (2003) |
| Carotenoid Isomerase | 1 | * | CRTISO  (*tangerine*) | Isaacson  *et al.* (2002) |
| Lycopene β-cyclase | 3 | LCY-B | B-CYC  (*Beta*) | Pecker *et al.* (1996); Ronen *et al.* (2000) |
| Lycopene ε-cyclase | 1 | CRTL-E | (*Delta*) | Ronen *et al.* (1999) |
| Carotene β-hydroxylase | 3 | CRTR-B1 | CRTR-B2  (*white flower*) | Galpaz *et al.* (2006) |
| Carotene ε-hydroxylase | 1 | CRTR-E^a,c^ |  | Tian *et al.* (2003) |
| Classification was determined from previously published data, digital expression data from the tomato expression database (<http://ted.bti.cornell.edu/>), or unpublished qRT-PCR data on full fruit development and ripening timecourse.  Tomato fruit color mutants are in ().  ‘*’ In photosynthetic tissue carotenoids undergo photoisomerization  ‘a’ *Arabidopsis* mutant – No tomato mutant published.  ‘b’ Maize mutant – No tomato mutant published.  ‘c’ Classification solely based on digital expression and qRT-PCR. | | | | |

| **Table S3. Primer list** | |
| --- | --- |
| **Primer Name** | **Primer Sequence** |
| ZDS-RNAi-FOR | GGGGACAAGTTTGTACAAAAAAGCAGGCTAGATGACAGACTGCAAATCATCC |
| ZDS-RNAi-REV | GGGGACCACTTTGTACAAGAAAGCTGGGTACAAAGTTCTTCTCAATGATTGATGAAGTAGC |
| AtZDS-OE-KpnI.for | GGTACCATGGCTTCTTCAGTCGTCTT |
| AtZDS-OE-XbaI.rev | TCTAGATTAGACCAGACTTAGCTCA |
| 35S-FOR | CCCAAGAAGGTTAAAGATGCAGTCA |
| 35S-REV | GCTTTGAAGACGTGGTTGGAACGTC |
| 35S-internal-FOR | AAGGAAGTTCATTTCATTTGGAGAG |
| AtZDS-internal-REV | TGGAGCTCCAACAGGAAACCGA |
| 35S-qPCR-for | CGGCAACAAACGGCGTTCCC |
| 35S-qPCR-rev | GTACGCGTCAGCTGCTGCTCT |
| PG-qPCR-for | ACGCCTCGTACATTCGAGATCGT |
| PG-qPCR-rev | TGCAATTCCAGAAGGTTAAGGCCTG |
| AtZDS-qRT-for | GCTGGTGAAGAACTAGCAGAGC |
| AtZDS-qRT-rev | CATCATTAGACCAGACTTAGCTC |
| SlZDS-qRT-for | GCTGCTGAGTTGAATGACATCTC |
| SlZDS-qRT-rev | GTGCGATGCCTAACTGAGTTG |
| CRTISO-qRT-for | CAGGACAAGGTGTTATAGCTGTA |
| CRTISO-qRT-rev | GAGCACTGTCCAGCACATCTGAT |
| FtsZ-FOR | CTGAAGAAGCAGCCGAACAA |
| FtsZ-REV | ACGCTCATCAACAACAGCAC |
| POR-B1-FOR (SGN-U577510) | CGGAAGAAAGTCATCCCTCGGAGCT |
| POR-B1-REV (SGN-U577510) | GGTTTTCTTTCCTGTCACAGGG |
| POR-B2-FOR (SGN-U580321) | CCAGTACCAGTAGTTCTACAGAC |
| POR-B2-REV (SGN-U580321) | GCTTTGCCCTTGGAGAAAGTAG |
| CAO-FOR | GGTGTGCAAAAGATATTGGGATC |
| CAO-REV | GTGCTCTGTCCCTCCAATTTCC |
| 18S-qRT-FOR | CGGAGAGGGAGCCTGAGAA |
| 18S-qRT-REV | CCCGTGTTAGGATTGGGTAATTT |
